# Primary Central Nervous System Lymphoma Presenting as Isolated Multiple Cranial Neuropathies: An Extremely Rare Case

**DOI:** 10.7759/cureus.41561

**Published:** 2023-07-08

**Authors:** Geoffrey B Lobban, Alex J Luke, Peter Basta, Katsiaryna Laziuk, Amandeep Kalra, Ashish Kulhari

**Affiliations:** 1 Department of Neurology, University of Missouri-Kansas City, Kansas City, USA; 2 Department of Neurosurgery, Research Medical Center, Kansas City, USA; 3 Department of Pathology, Research Medical Center, Kansas City, USA; 4 Department of Neuro-oncology, Research Medical Center, Kansas City, USA; 5 Department of Neurology, University of Missouri Kansas-City School of Medicine, Kansas City, USA; 6 Department of Neurology, Kansas City University of Medicine and Biosciences, Kansas City, USA; 7 Department of Neurology, Research Medical Center, Kansas City, USA

**Keywords:** trigeminal nerve biopsy, trigeminal neuralgia, bells palsy, isolated cranial nerve palsy, cns lymphoma

## Abstract

Primary central nervous system lymphoma (PCNSL) is an uncommon malignancy of B-cell origin that typically involves the brain, eyes, and spinal cord without systemic spread. PCNSL typically involves the cerebral hemispheres, basal ganglia, or periventricular region. Isolated leptomeningeal PCNSL without any evidence of parenchymal involvement is very rare. We present a very unusual case of PCNSL presenting as persistent bilateral Bell's palsy and trigeminal neuralgia with magnetic resonance imaging (MRI) brain showing significantly hypertrophied enhancing bilateral facial and trigeminal nerves.

## Introduction

Primary central nervous system lymphoma (PCNSL) is a rare, B-cell-mediated, extranodal, non-Hodgkin lymphoma confined to the brain, eyes, and spinal cord without systemic involvement [1]. PCNSL represents about 4% of all intracranial neoplasms [1]. The isolated leptomeningeal PCNSL without any evidence of parenchymal involvement is very rare, occurring in only 7% of PCNSL cases [2]. PCNSL can affect both immunocompromised and immunocompetent hosts, including those with HIV, transplant recipients, or those on immunotherapies. Since the highly active antiretroviral therapy's (HAART) introduction, PCNSL has had a much higher frequency in immunocompetent individuals [3]. PCNSL typically presents with focal neurological deficits (56-70%), neuropsychiatric symptoms (32-43%), symptoms of increased intracranial pressure (32-33%), and seizures (11-14%) [1,4]. Magnetic resonance imaging (MRI) of the brain typically shows supratentorial (87%), homogeneously enhancing, solitary mass lesions (66%) in fronto-parietal lobes (39%) [1,5]. Stereotactic biopsy is the procedure of choice to establish a diagnosis [1]. PCNSL often demonstrates a response to chemotherapy and radiotherapy despite higher mortality than most peripheral lymphomas [1,6-8]. We present a very unusual case of PCNSL presenting with isolated persistent bilateral Bell's palsy and trigeminal neuralgia with MRI brain showing significantly hypertrophied enhancing bilateral facial and trigeminal nerves, treated with high-dose methotrexate and rituximab.

## Case presentation

**Figure 1:**
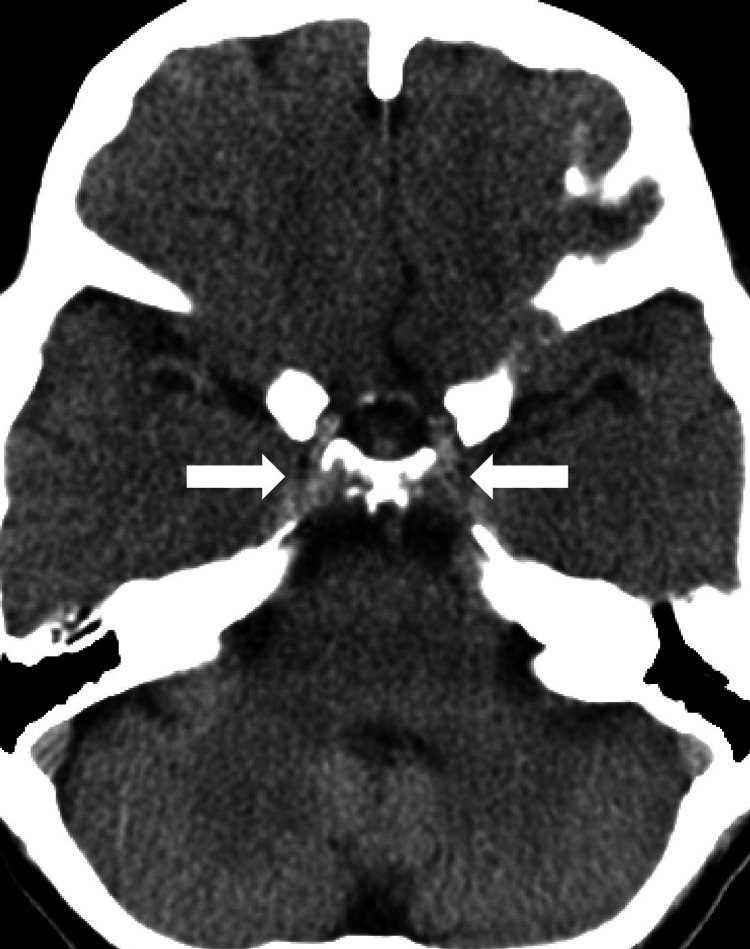
Computed tomography of the head showing fullness in the bilateral cavernous sinus.

A 67-year-old woman with no significant past medical history except recently diagnosed bilateral Bell's palsy and trigeminal neuralgia, was admitted to our hospital for generalized weakness and malaise due to poor oral intake. The patient was undergoing outpatient diagnostic work-up for persistent Bell's palsy (diagnosed four months prior) and trigeminal neuralgia (diagnosed two months prior). The patient's neurological examination showed bilateral lower motor neuron facial palsy and decreased sensation to light touch and pin-pricks on the face; the rest of the exam was normal. The computed tomography (CT) of the head showed fullness in the bilateral (left greater than right) cavernous sinus (Figure 1).

**Figure 2:**
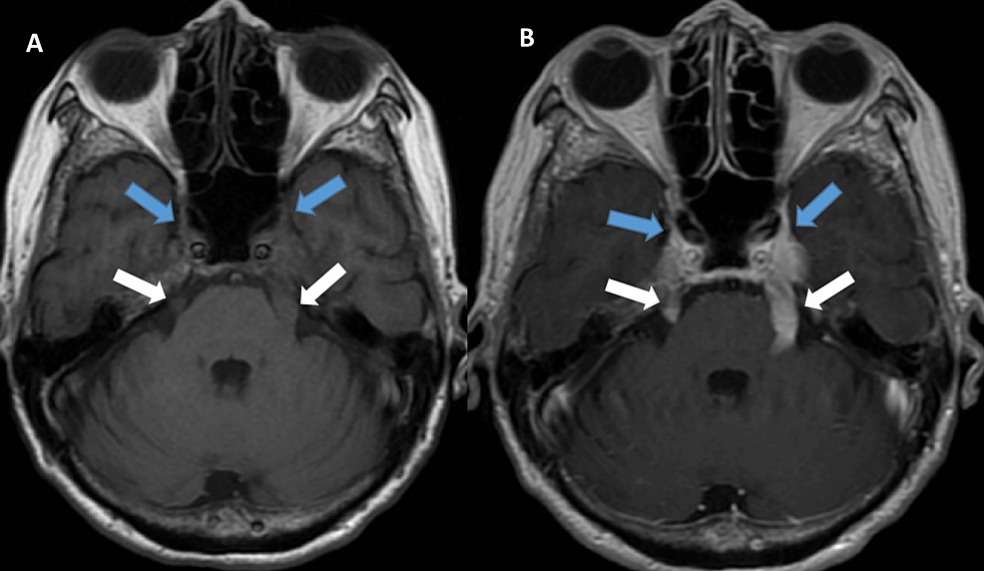
(A) MRI of the brain without contrast showing hypertrophied bilateral trigeminal (white arrows) and facial nerves (blue arrows). (B) MRI of the brain with contrast showing significant enhancement of bilateral trigeminal (white arrows) and facial nerves (blue arrows). MRI: magnetic resonance imaging.

CT angiography (CTA) of the head and neck did not show any vascular abnormality. MRI of the brain with/without contrast showed enhancing, significantly hypertrophied bilateral trigeminal and facial nerves (Figure 2).

**Figure 3:**
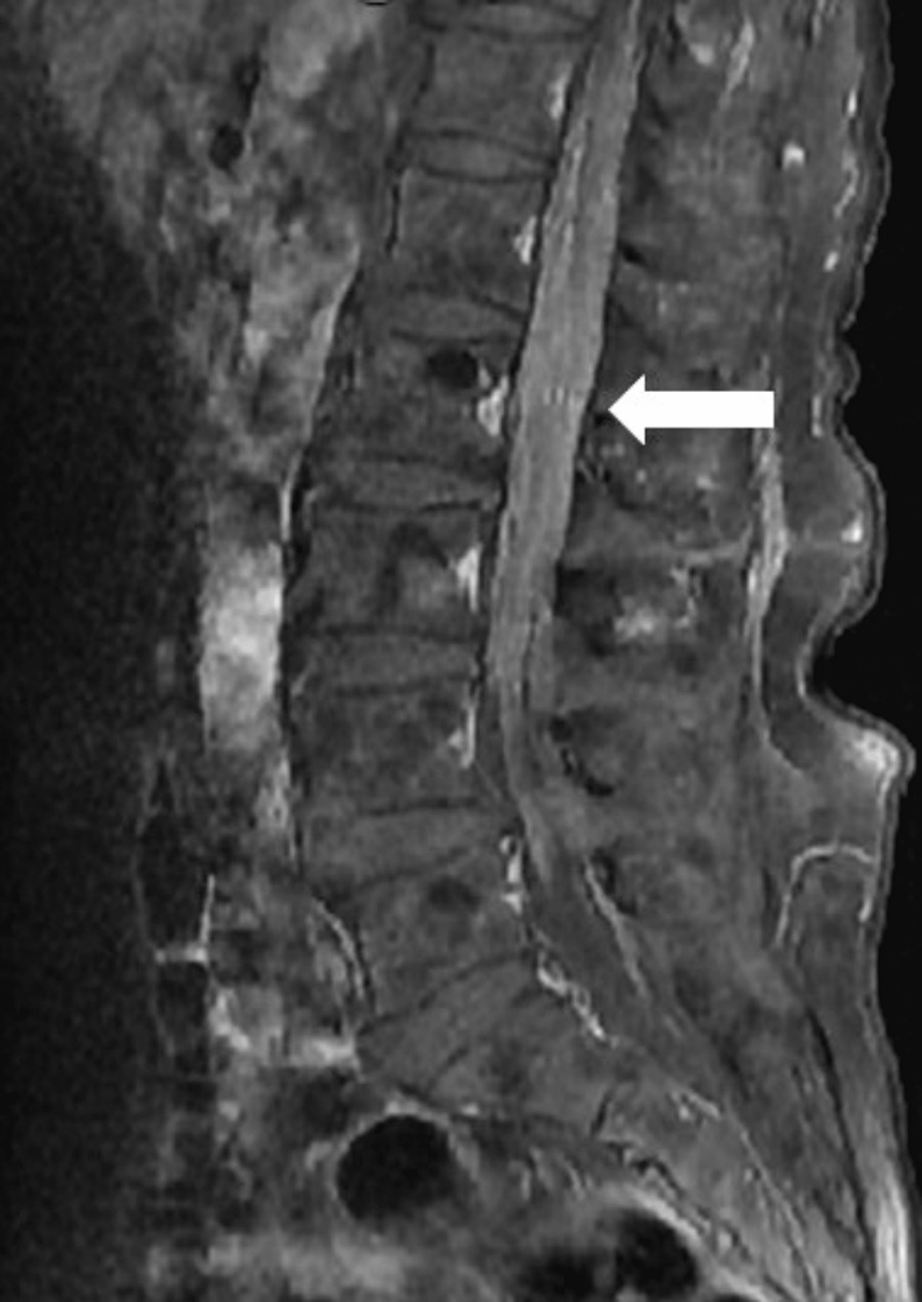
Lumbar MRI with contrast showing leptomeningeal enhancement of the cauda equina. MRI: magnetic resonance imaging.

The patient underwent a lumbar puncture twice for further work-up. Basic cerebrospinal fluid (CSF) studies revealed lymphocytic pleocytosis (144 nucleated cells with 88% lymphocytes; 96 nucleated cells with 98% lymphocytes) with significantly elevated protein (279; 455) and normal glucose (85; 81). CSF cytology and flow cytometry showed the predominance of T cells without a monotypic B-cell population, which was inconclusive of lymphoma. Infectious work-up, including meningitis encephalitis PCR panel, venereal disease research laboratory (VDRL), and cryptococcal antigen, was negative. CSF lactate dehydrogenase (LDH) and angiotensin-converting enzyme (ACE) were within normal limits. Serum antinuclear antibody (ANA), erythrocyte sedimentation rate (ESR), and C-reactive protein (CRP) were unremarkable. Lumbar MRI with contrast showed leptomeningeal enhancement of the cauda equina (Figure 3).

**Figure 4:**
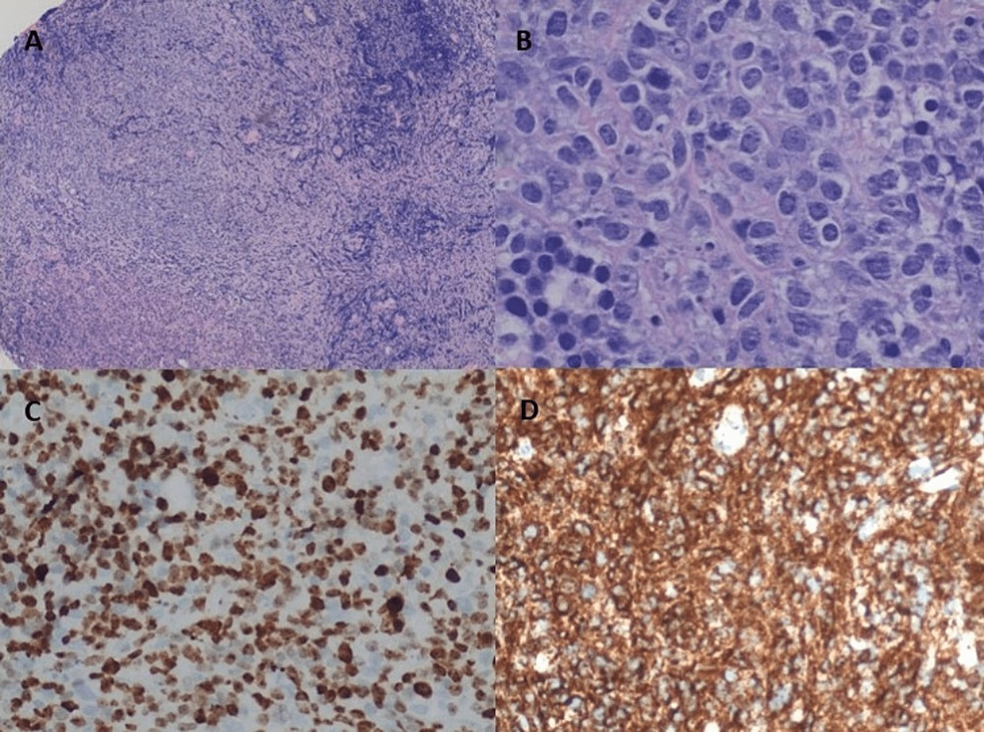
(A) H&E staining (40×) showing monotonous proliferation of large lymphoid cells. (B) H&E staining (400×) showing neoplastic cells with large nuclei and prominent nucleoli. (C) Proliferation index KI-67. (D) CD20 immunohistochemical stain. H&E: hematoxylin and eosin.

Due to inconclusive CSF results, neurosurgery was consulted for a trigeminal nerve biopsy. The patient underwent a left trigeminal nerve biopsy that confirmed diffuse large B-cell lymphoma. Histologic sections demonstrated the monotonous proliferation of large lymphoid cells with irregular nuclear contours and prominent nucleoli. CD 20 and PAX5 staining confirmed B cells positive for MUM1, BCL2, and BCL6 markers, with a KI-67 proliferation index of 80% (Figure 4). The patient started on a high-dose methotrexate and rituximab chemotherapy regimen. 

## Discussion

While the symptoms of PCNSL are wide-ranging, including symptoms such as nausea, seizure, headache, focal weakness, diplopia, hearing loss, and altered mental status, it is exceedingly rare to present as a cause of isolated facial paralysis [1]. The lack of response to traditional treatment of Bell’s palsy alongside the development of additional symptoms related to other cranial nerve functions should lead a practitioner to consider other more diffuse CNS diseases. It is also worthwhile reiterating that while immunosuppression is recognized as a risk factor for PCNSL, most cases are now among the immunocompetent [3], such as this case.

Perhaps the most interesting findings in this case pertains to brain imaging. Typically, MRI brain shows supratentorial (87%), homogeneously enhancing, solitary mass lesion (66%) in fronto-parietal lobes (39%), with multiple masses seen in 20-40% of the PCNSL cases [1,5]. The isolated leptomeningeal PCNSL without any evidence of parenchymal involvement is very rare, occurring in only 7% of PCNSL cases [2], such as this case.

Despite of inconclusive CSF studies, we proceeded with a trigeminal nerve biopsy due to high suspicion of malignancy. Ultimately, a biopsy confirmed the diagnosis of diffuse B cell lymphoma. High-dose (HD) methotrexate therapy has been the standard of care almost without exception alongside additional chemotherapeutic agents such as rituximab [9,10].

Multiple medical teams, including internal medicine, neurology, neuro-oncology, neurosurgery, and neuropathology, were involved in our patient's care, emphasizing the importance of a multidisciplinary approach in treating complex neurological conditions.

## Conclusions

We present an extremely rare case of isolated leptomeningeal primary central nervous system lymphoma presenting with progressive multiple cranial neuropathies, highlighting the atypical brain imaging findings and emphasizing the importance of pursuit of biopsy when clinical suspicion is high. Given the rarity of this disease, we believe our case will robust the literature on isolated leptomeningeal PCNSL.
